# The impact of homologous recombination deficiency on the prognosis of epithelial ovarian cancer

**DOI:** 10.1002/ctm2.70143

**Published:** 2024-12-26

**Authors:** Haiqi Su, Yinan Wang, Xiaopei Chao, Huanwen Wu, Yan You, Shuru Zhao, Feiyue Wang, Bao Sun, Zhen Zhang, Ming Wu, Zicheng Zhao, Lei Li

**Affiliations:** ^1^ Department of Obstetrics and Gynecology Peking Union Medical College Hospital Beijing China; ^2^ National Clinical Research Center for Obstetric & Gynecologic Diseases Beijing China; ^3^ State Key Laboratory for Complex Severe and Rare Diseases Peking Union Medical College Hospital Beijing China; ^4^ Department of Obstetrics and Gynecology Peking University Shenzhen Hospital, 1120 Lianhua Road Shenzhen China; ^5^ School of Medicine Southern University of Science and Technology, 1088 Xueyuan Avenue Shenzhen China; ^6^ Department of Pathology Peking Union Medical College Hospital Beijing China; ^7^ Shenzhen Byoryn Technology Co., Ltd, No. 14, Jinxiu Road Shenzhen China; ^8^ BGI Genomics, BGI‐Shenzhen Shenzhen China

1

Dear Editor,

Homologous recombination deficiency (HRD) is a potential biomarker for predicting the efficacy of platinum‐based therapies, PARP inhibitor (PARPi) therapies and overall survival outcomes in epithelial ovarian cancer (EOC).^1^, ^2^ Nevertheless, the methodologies for HRD detection and the optimal timing for HRD assessment in tumour samples remain contentious and are infrequently examined within the Chinese EOC patient population.^3^, ^4^, ^5^, ^6^, ^7^ In this study, we enrolled a cohort of 201 Chinese EOC patients who underwent a modified HRD test. This test incorporated adjustments for tumour purity and ploidy to refine the HRD score across various time intervals post‐diagnosis, spanning from 1 day to 10 years. Our objective was to evaluate the feasibility of employing modified HRD genomic scar assays and to assess the impact of the timing of the test.

Between July 2007 and November 2021, a total of 233 patients diagnosed with epithelial ovarian cancer, fallopian tube cancer, or primary peritoneal cancer were enrolled in the study. These patients underwent first‐line chemotherapy regimens containing platinum and were monitored until 1 March 2022. Of these, 32 patients were excluded due to the absence of HRD score testing or genetic testing, resulting in 201 eligible patients included in the final analysis (Figure S1). The HRD score was determined using a genomic scar analysis (GSA) algorithm,^8^ which assessed the loss of heterozygosity (LOH),^9^ telomeric allelic imbalance (TAI) and large‐scale state transitions (LST),^10^ while also considering tumour purity and ploidy (Table S1). A comprehensive analysis was conducted to assess mutation information across 68 genes, comprising 57 genes associated with homologous recombination repair (HRR), such as BRCA1/2, PALB2 and ATM, as well as 11 genes recurrently implicated in EOC, as detailed in Table S2. The process for HRD assessment is illustrated in Figure S2. Table S3 presents the quality control of genomic scar analyses, Table S4 details the somatic mutations in 68 genes across 124 patients, and Table S5 outlines the germline mutations in 68 genes from 201 patients. In summary, a patient was classified as HRD positive if they exhibited HRD scores of 30 or higher (indicative of a high HRD score) and/or possessed mutations in BRCA1/2 genes, characterized as germline mutations of class 4/5 or somatic mutations of tier I/II. Conversely, patients were considered to have homologous recombination non‐deficiency if their HRD scores were below 30 (indicative of a low HRD score) and they lacked mutations in BRCA1/2 genes.

Among 201 patients, the median age at diagnosis was 55 years (range 24–82). Most had high‐grade serous carcinoma (171, 85.07%) or advanced‐stage disease (130, 64.68%). Over half (119, 59.20%) had interval debulking surgery, while the rest had primary debulking or staging surgery. All received first‐line treatment. After a median follow‐up of 40 months, 83.58% were sensitive to chemotherapy and 13.43% were resistant. Recurrence and death rates were 80.01% and 36.32%, respectively. Twenty‐six patients underwent first‐line PARPi maintenance therapy. (Table 1 and Figure 1). A total of 3702 nonsynonymous germline and 263 nonsynonymous somatic coding mutations were found in 97.51% (196/201) and 82.26% (102/124) of patients, respectively. HRD status was evaluated using the HRD score and BRCA1/2 mutations. Among the patients, 88 (43.78%) had high HRD scores (≥30, Figure 2A). Of the 113 patients with low HRD scores (<30), 23 had significant germline or tier I/II somatic BRCA1/2 mutations. Two patients had both germline and somatic BRCA1 mutations, and four had both in BRCA2. A total of 111 patients tested HRD positive, while 52 had undetermined HRD status due to insufficient somatic testing and VUS. Thirty‐eight patients were HRD negative (Table S1). Of the patients, 173 were tested for HRD scores within 6 months of diagnosis, and 28 were tested after 6 months.

**TABLE 1:** The clinical and pathological characteristics of the participants.

Characteristic (*n* = 201)	
Age‐years	
Median	55
Range	24–82
**Epithelial ovarian cancer type**	
Ovary cancer	190 (94.53%)
Fallopian tube cancer	7 (3.48%)
Primary peritoneal cancer	4 (1.99%)
**Histological type**	
Serous carcinoma	174 (86.57%)
Mucinous carcinoma	4 (1.99%)
Endometrioid carcinoma	7 (3.48%)
Clear cell carcinoma	11 (5.34%)
Carcinosarcoma	3 (1.49%)
Mixed types	2 (1.00%)
**FIGO stage**	
I	21 (10.45%)
II	25 (12.44%)
III	127 (63.18%)
IV	28 (13.93%)
**First‐line surgery**	
IDS^*^	119 (59.20%)
PDS^*^ or staging surgery	81 (40.30%)
No	1 (0.50%)
**Surgery residual**	
R0^#^	82 (40.80%)
R1^#^	19 (9.45%)
R2^#^	6 (2.99%)
Unknown	94 (46.77%)
**First‐line treatment**	
Paclitaxel/carboplatin	181 (90.05%)
Paclitaxel/cisplatin	16 (7.96%)
Carboplatin	1 (0.50%)
Liposomal doxorubicin/carboplatin	1 (0.50%)
Paclitaxel/nedaplatin	1 (0.50%)
Albumin‐bound paclitaxel/cisplatin	1 (0.50%)
**Combinations with bevacizumab in first‐line chemotherapy**	
Yes	27 (13.43%)
No	174 (86.57%)
**Platinum sensitivity**	
Sensitivity	168 (83.58%)
Resistance	27 (13.43%)
Refractory	6 (2.99%)
**PARPi therapy after first‐line chemotherapy**	
Niraparib	12 (5.97%)
Olaparib	14 (6.97%)
No	175 (87.06%)
**Personal cancer history**	
Yes	14 (6.97%)
No	187 (93.03%)
**Family history of cancer**	
Yes	88 (43.78%)
No	111 (55.22%)
Unknown	2 (1.00%)
**History of alcohol consuming**	
Yes	3 (1.49%)
No	198 (98.51%)
**History of tobacco consuming**	
Yes	3 (1.49%)
No	198 (98.51%)

* IDS: interval debulking surgery; PDS: primary debulking surgery.

^#^ The tumour status following surgery was defined using the residual tumour (R) classification: R0, no residual tumour; R1, greatest diameter of residual tumour *x* ≤ 1 cm; R2, greatest diameter of residual tumour > 1 cm.

**FIGURE 1:**
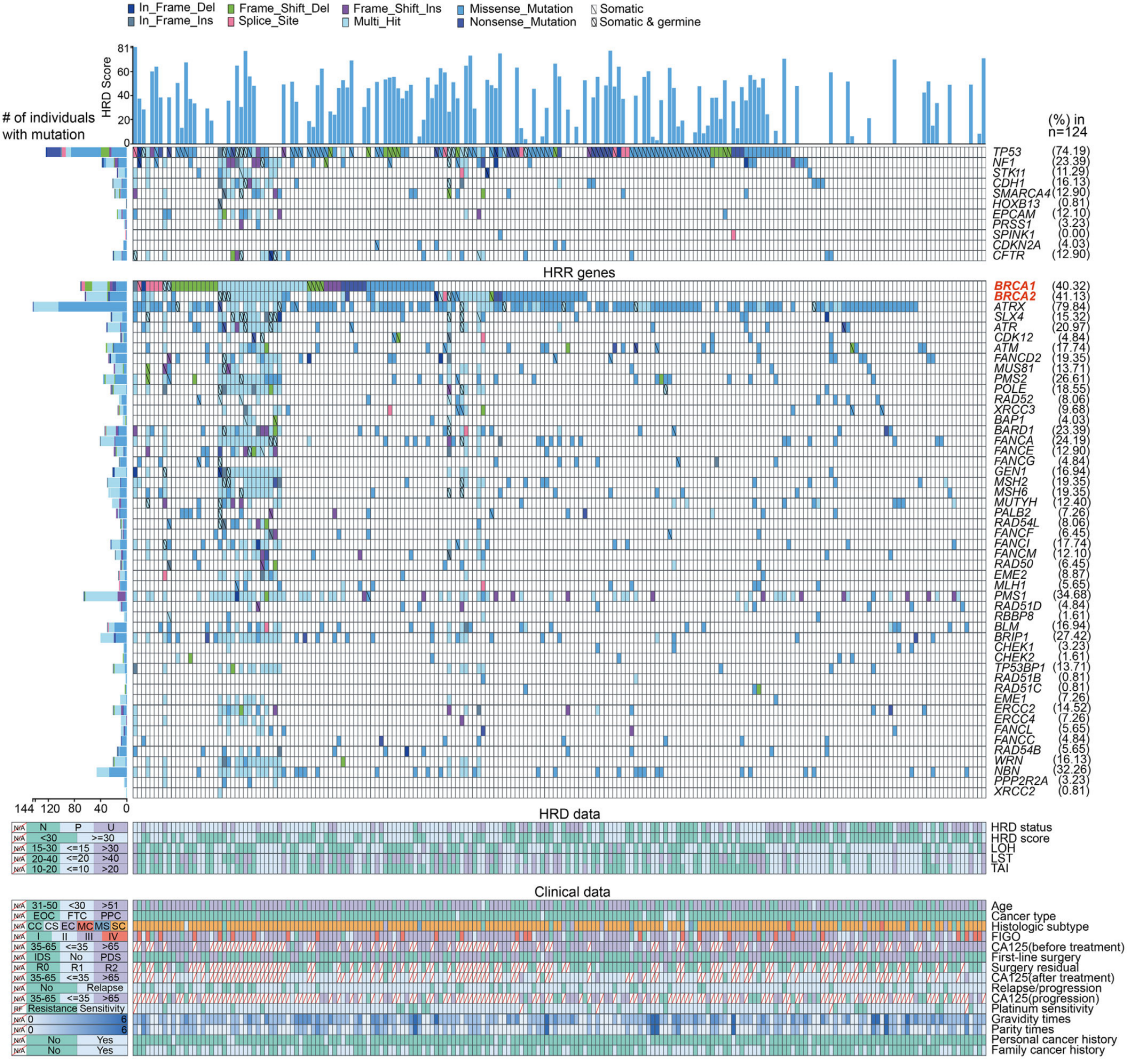
Clinical characteristics and variants of homologous recombination genes and recurrent genes from 201 patients with epithelial ovarian cancer (EOC). The right percentage was the sum of each gene's somatic and germline variant frequency in 124 patients with germline and somatic genetic tests. Put differently, variants from another 77 patients only with germline genetic tests were shown but not counted in the variant frequency. Somatic mutation is annotated with a diagonal line. Coexisting germline and somatic mutations are annotated with circles and diagonal lines inside the square. The middle panel shows the homologous recombination deficiency (HRD)‐related evaluation metrics. The level of genomic instability of each sample is depicted by HRD status. The bottom panel shows key clinic pathological characteristics. TAI, telomeric allelic imbalance; HRD, homologous recombination deficiency; LST, large‐scale state transition; LOH, loss of heterozygosity; CA125, cancer antigen 125; RF: refractory.

**FIGURE 2:**
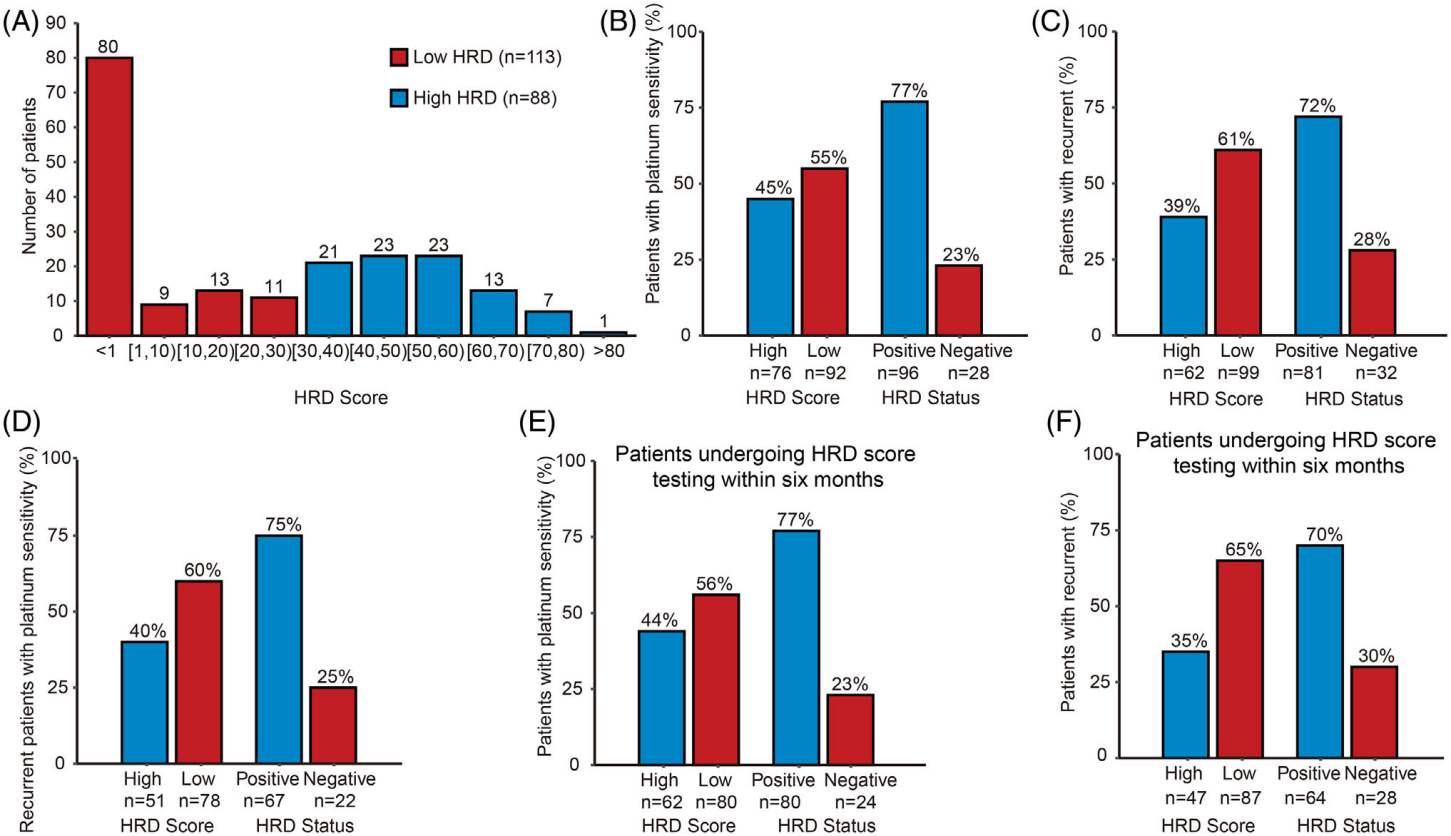
Homologous recombination deficiency (HRD) score distribution and relative response rates for patients with epithelial ovarian cancer (EOC). (A) HRD score distribution in the EOC patients. Relative response rates for patients with platinum sensitivity (B), recurrent patients (C), recurrent patients with platinum sensitivity (D), platinum−sensitive patients undergoing HRD score testing within 6 months after diagnosis (E), recurrent patients undergoing HRD score testing within 6 months after diagnosis stratified by HRD score and HRD status (F).

HRD status was linked to the response to platinum‐based chemotherapy, with significant associations for all patients (*p* = .02) and for those tested within 6 months of diagnosis (*p* = .01) (Table S6 and Figure 2B,E). The HRD‐positive group (*n* = 108) had an 88.89% platinum sensitivity rate. Additionally, the HRD score was related to relapse, independent of platinum therapy response or testing time (*p* = .003 for all patients, *p* = .01 for platinum‐sensitive patients and *p* = .001 for those tested within 6 months, Table S6 and Figure 2C,D,F). No survival time difference was found between high and low HRD score groups for all patients (overall survival [OS] *p* = .085, progression‐free survival [PFS] *p* = .082, Figure S3). However, among patients tested within 6 months of diagnosis, the high HRD score group (*n* = 101) showed better OS (*p* = .022) and PFS (*p* = .023) than the low HRD score group (*n *= 72, Figure 3A,B). At 3 years, patients with high HRD scores had OS and PFS rates of 74.83% and 67.99%, compared to 59.69% and 51.2% for those with low scores. In high‐grade serous carcinoma, higher HRD scores were linked to better OS (*p* = .015) and PFS (*p* = .018, Figure 3C,D). Three‐year and 5‐year survival rates were 74.02% and 62.55% for high HRD scores, and 58.72% and 27.36% for low scores. High HRD scores correlated with significantly improved OS and PFS compared to low scores (*p* = .032 for OS, *p* = .008 for PFS) in 26 patients on first‐line PARPi maintenance therapy. At three years, PFS was 84.00% for high HRD scores and 36.32% for low scores. Median HRD scores were 8.45, 18.66, 17.58 and 10.01 in patients with FIGO stages I—IV, respectively, and no significant difference was found (*p* = .67, Figure S4).

**FIGURE 3:**
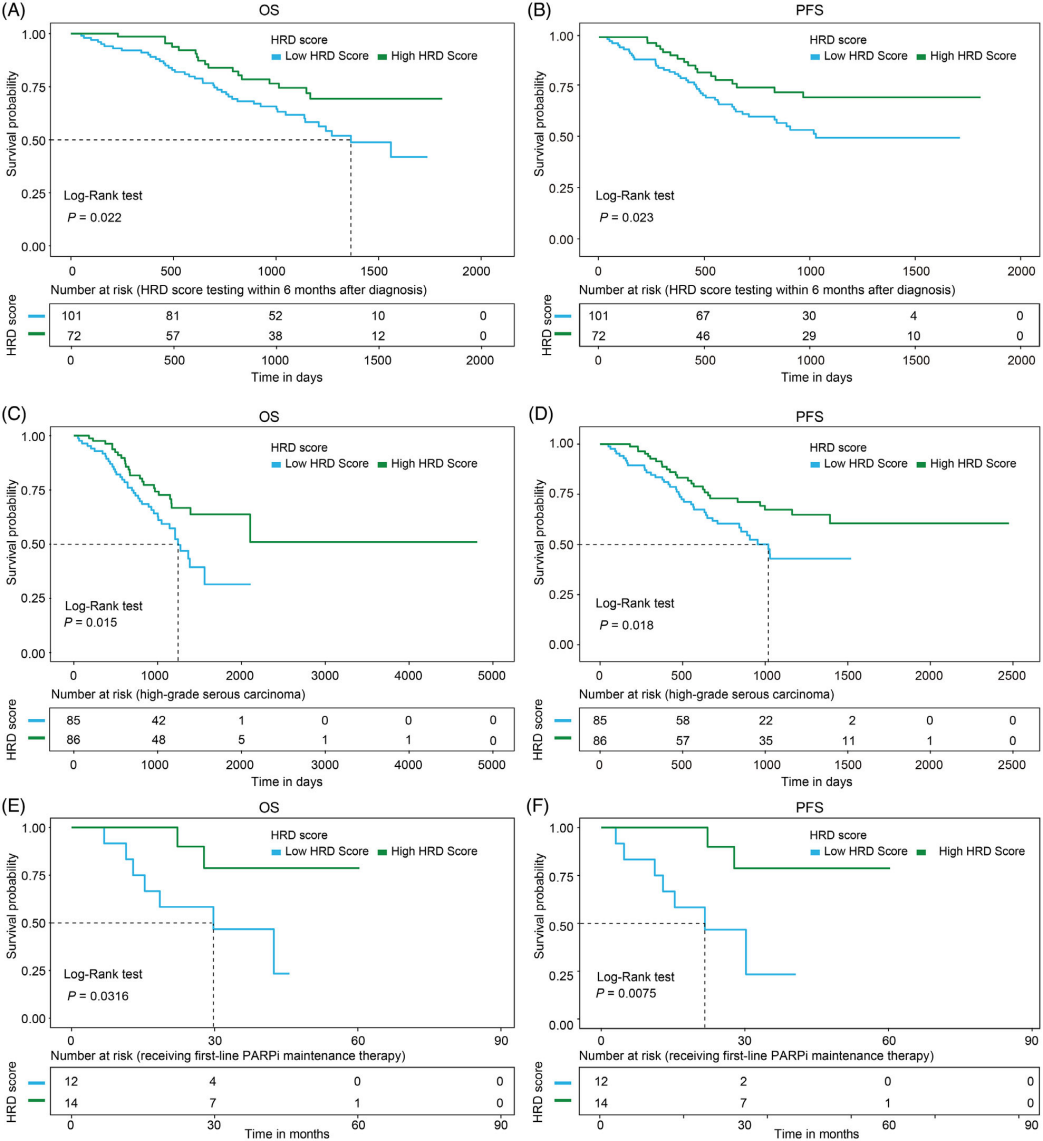
Overall survival (OS) and progression‐free survival (PFS) by homologous recombination deficiency (HRD) score. (A) OS of patients undergoing HRD score testing within 6 months after diagnosis. (B) PFS of patients undergoing HRD score testing within 6 months after diagnosis. (C) OS of patients with high‐grade serous carcinoma. (D) PFS of patients with high‐grade serous carcinoma. (E) OS of patients receiving first‐line PARP inhibitor (PARPi) maintenance therapy. (F) PFS of patients receiving first‐line PARPi maintenance therapy.

Our study shows that HRD status, identified through genomic scars and HRR gene mutations, predicts platinum sensitivity in EOC. High HRD scores correlate with improved OS and PFS in patients on first‐line PARPi maintenance. Moreover, those with high‐grade serous carcinoma and HRD positivity had notably higher three‐ and five‐year survival rates, along with extended OS and PFS. Interestingly, a low HRD score was linked to a higher likelihood of relapses, particularly in patients tested within 6 months of diagnosis. Limitations of our study include: 52 patients lacked tissue samples for somatic mutation detection, leaving their HRD status undetermined, and the exclusive focus on Chinese EOC patients may limit the findings' applicability to other populations.

In summary, we adjusted the HRD score using tumour purity and ploidy, resulting in a model that effectively predicts platinum chemotherapy and PARPi efficacy, recurrence and survival, particularly for patients tested within 6 months of diagnosis. Our results aid genetic risk assessment, treatment decisions and prediction prognosis.

## AUTHOR CONTRIBUTIONS

Haiqi Su, Lei Li, Ming Wu and Zhen Zhang conceived of the original idea for the study, interpreted the results, carried out the statistical analysis, edited the paper and was the overall guarantor. Lei Li, Yinan Wang and Bao Sun obtained ethical approval, contributed to the preparation of the data set, interpreted results and contributed to drafts of the paper. Xiaopei Chao, Shuru Zhao, Zhen Zhang and Feiyue Wang contributed to the study design, and interpretation of results and commented on drafts of the paper. Huanwen Wu and Yan You conducted the pathological evaluation and reviewed the original materials. All authors have approved the final version of the manuscript.

## CONFLICT OF INTEREST STATEMENT

The authors declare no conflict of interest.

## PATIENT CONSENT STATEMENT

Informed consent was obtained from the subjects prior to participating in the study.

### ETHICS STATEMENT

The Institutional Review Board of Peking Union Medical College Hospital has approved this study (No.JS‐2441). The registration number is NCT04651920 (*clinicaltrials.gov*, registered on 26 November 2020).

## Supporting information



FIGURE S1 Flow diagram for the study cohort selection.

FIGURE S2 Adjudication of HRD status.

FIGURE S3 Overall survival and progression‐free survival by the HRD score for all patients.

FIGURE S4 Median HRD score with FIGO stages I—IV

TABLE S1 Clinical characteristics of 201 patients.
TABLE S2 Targeted sequencing of HR‐related genes.
TABLE S3 Quality control of genomic scar analyses.
TABLE S4 Somatic mutations in 68 genes of 124 patients.
TABLE S5 Germline mutations in 68 genes of 201 patients.
TABLE S6 HRD score and HRD status associations with response to platinum‐containing therapy and relapse

Supporting Information

## Data Availability

All clinical data and variation data of this study have been contained in the supplement file.
