# Supplementary figures and images for: The impact of homologous recombination deficiency on the prognosis of epithelial ovarian cancer
Source: Clin Transl Med. 2024 Dec 26;15(1):e70143. doi: 10.1002/ctm2.70143 (PMC11670307; doi:10.1002/ctm2.70143)

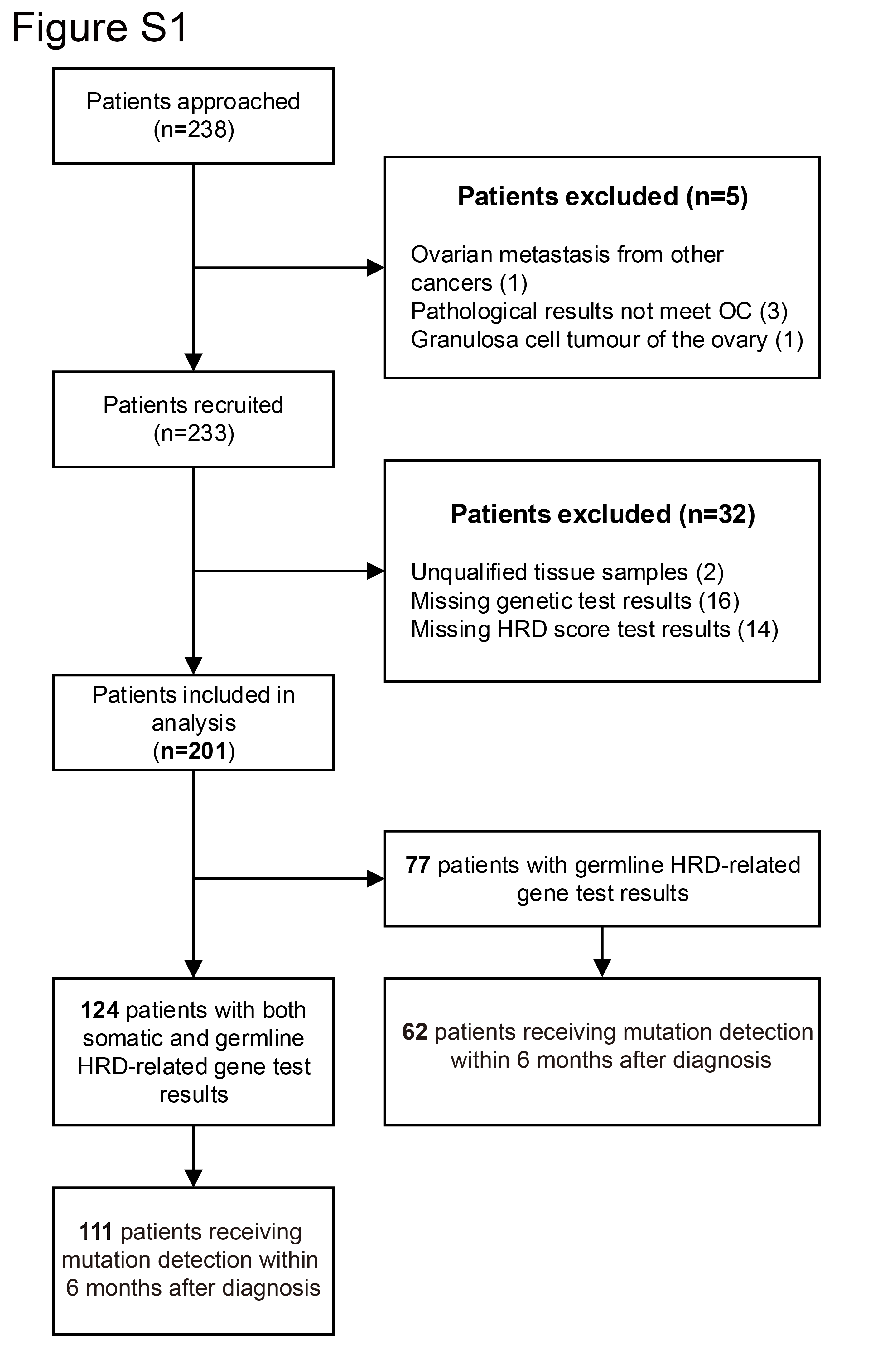

Supplement: Supplementary file 1 — FIGURE S1 Flow diagram for the study cohort selection. [file CTM2-15-e70143-s004.tif]

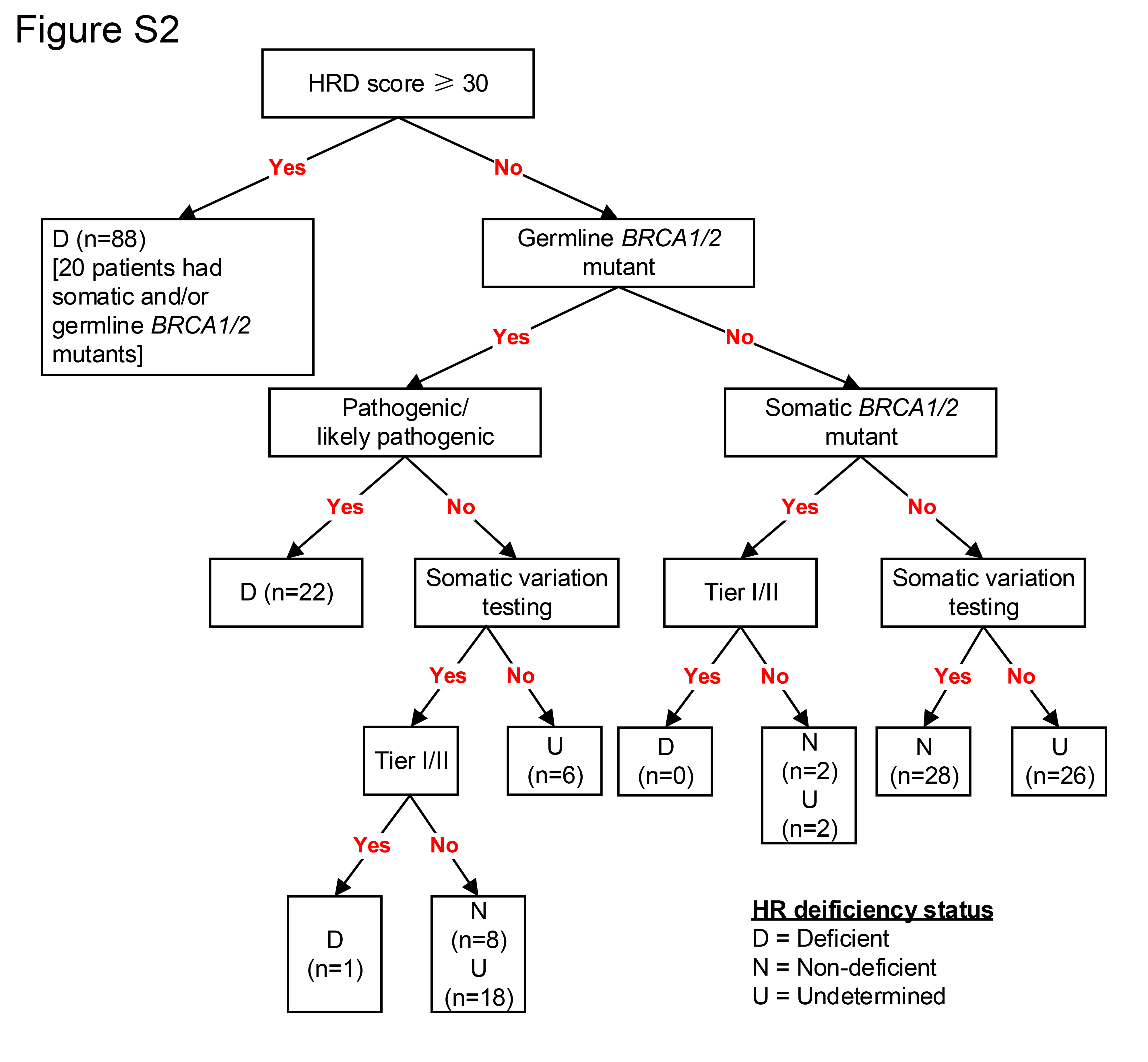

Supplement: Supplementary file 2 — FIGURE S2 Adjudication of HRD status. [file CTM2-15-e70143-s006.tif]

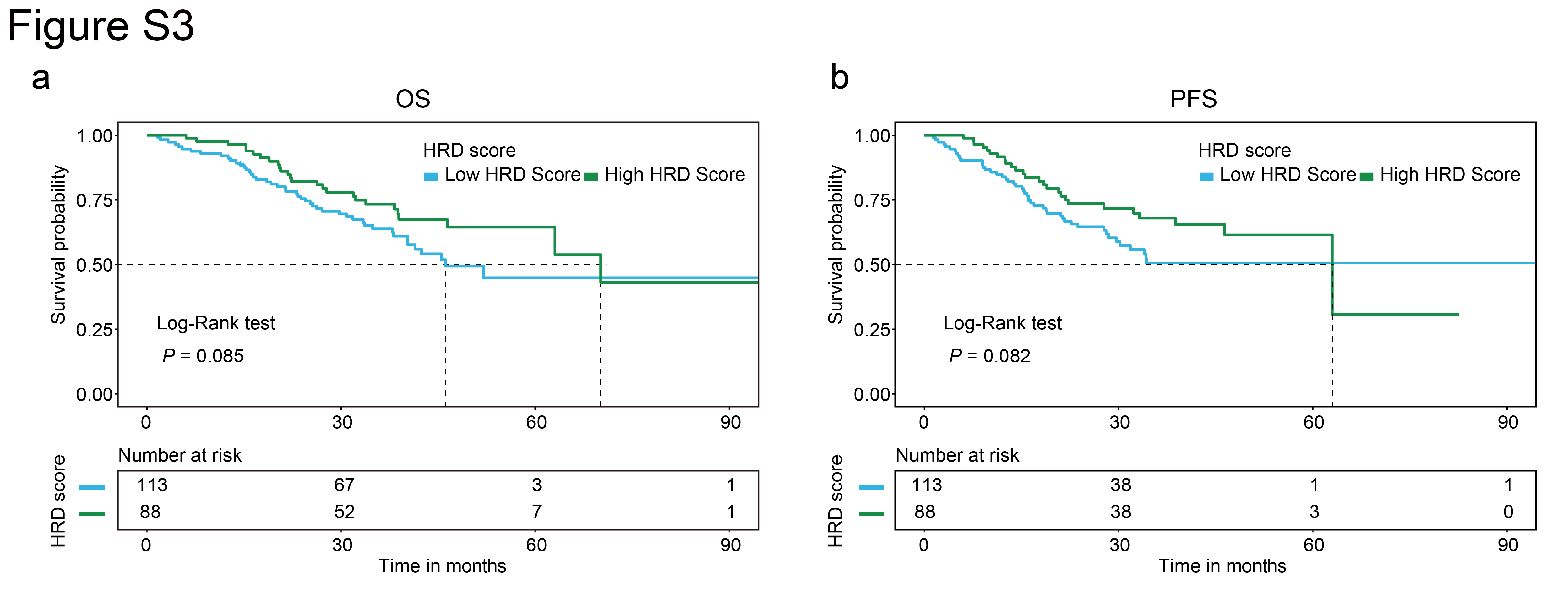

Supplement: Supplementary file 3 — FIGURE S3 Overall survival and progression‐free survival by the HRD score for all patients. [file CTM2-15-e70143-s005.tif]

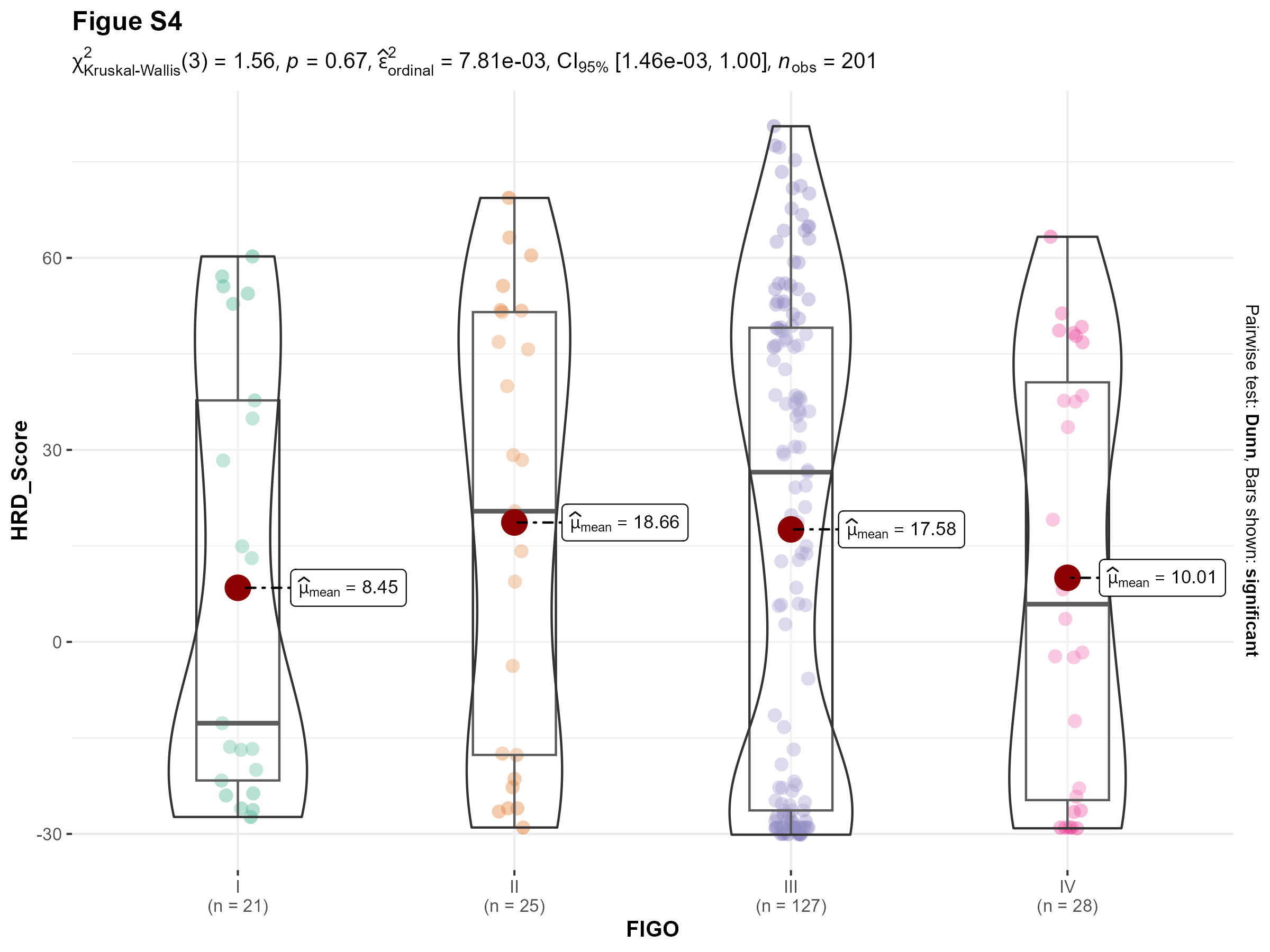

Supplement: Supplementary file 4 — FIGURE S4 Median HRD score with FIGO stages I—IV [file CTM2-15-e70143-s003.tif]
