# Supplementary material for: The impact of homologous recombination deficiency on the prognosis of epithelial ovarian cancer
Source: Clin Transl Med. 2024 Dec 26;15(1):e70143. doi: 10.1002/ctm2.70143 (PMC11670307; doi:10.1002/ctm2.70143)
Supplement: Supplementary file 6 — Supporting Information [file CTM2-15-e70143-s001.docx]

**Table S6. HRD score and HRD status associations with response to platinum-containing therapy and relapse**

|  |  | **n** | **HRD Score** | | **p** | **n** | **HRD status** | | **p** |
| --- | --- | --- | --- | --- | --- | --- | --- | --- | --- |
|  |  |  | **≥30** | **<30** |  |  | **Positive** | **Negative** |  |
| For all patients (n=201) | | | | | | | | | |
| Platinum sensitivity | Sensitivity | 168 | 76 (45.24%) | 92 (54.76%) | 0.25 | 124 | 96(77.42%) | 28 (22.58%) | 0.02 |
|  | Resistance | 27 | 9 (33.33%) | 18 (66.67%) |  | 22 | 12 (54.55%) | 10 (45.45%) |  |
| Relapse | Yes | 161 | 62 (38.5%) | 99 (61.5%) | 0.003 | 113 | 81 (71.68%) | 32 (28.32%) | 0.16 |
|  | No | 41 | 26 (65%) | 14 (35%) |  | 36 | 30 (83.33%) | 6 (16.67%) |  |
| For patients characterized as platinum-sensitive (n=168) | | | | | | | | | |
| Relapse | Yes | 129 | 51 (39.53%) | 78 (60.47%) | 0.01 | 89 | 67 (75.28%) | 22 (24.72%) | 0.36 |
|  | No | 39 | 25 (64.1%) | 14(35.9%) |  | 35 | 29 (82.86%) | 6 (17.14%) |  |
| For patients undergoing HRD score testing within six months after diagnosis (n=173) | | | | | | | | | |
| Platinum sensitivity | Sensitivity | 142 | 62(43.66%) | 80(56.34%) | 0.14 | 104 | 80(76.92%) | 24(23.08%) | 0.01 |
|  | Resistance | 25 | 7(28%) | 18(72%) |  | 20 | 10(50%) | 10(50%) |  |
| Relapse | Yes | 134 | 47(35.07%) | 87(64.93%) | 0.001 | 92 | 64(69.57%) | 28(30.43%) | 0.13 |
|  | No | 39 | 25(64.1%) | 14(35.9%) |  | 35 | 29(82.86%) | 6(17.14%) |  |
